# Enrichment of short-chain fatty acid-producing bacteria by pH-responsive sodium alginate and chitosan-encapsulated quercetin

**DOI:** 10.3389/fmicb.2025.1594012

**Published:** 2025-08-06

**Authors:** Qianyu Bai, Zhongling Zhao, Yujing Duan, Runqiu Cai, Yinzhu Chen, Chaoyu Zhou, Xinyuan Tian, Yifei Yang, Haiyan Wu, Mingju Li, Jia You, Qingyi Song, Hong Dong, Tianlong Liu

**Affiliations:** ^1^State Key Laboratory of Veterinary Public Health and Safety, College of Veterinary Medicine, China Agricultural University, Beijing, China; ^2^Beijing Key Laboratory of Traditional Chinese Veterinary Medicine, Beijing University of Agriculture, Beijing, China; ^3^Beijing Wildlife Park, Beijing, China; ^4^Yantai Animal Disease Control Center, Yantai, China; ^5^Yantai Agricultural Technology Extension Center, Yantai, China; ^6^Century Muming Feed Research Institute, Beijing, China

**Keywords:** sodium alginate, chitosan, ulcerative colitis, target delivery, mucosal barrier restoration, short-chain fatty acids

## Abstract

**Introduction:**

Conventional approaches to treat ulcerative colitis (UC) focus on suppressing excessive inflammation and immune responses. Nevertheless, these treatments fail to address gut dysbiosis or restore the intestinal mucosal barrier effectively. Regulating the intestinal microenvironment may be pivotal to more effective therapies for UC.

**Methods:**

Herein, oral colon-targeted microspheres, sodium alginate-chitosan-encapsulate quercetin (SA-Q-CS MPs), were developed. The stability and pH responsiveness of SA-Q-CS MPs were explored. Therapeutic effects were assessed in dextran sulfate sodium (DSS)-induced ulcerative colitis in female C57BL/6 mice via 16S ribosomal RNA (rRNA) gene sequencing, Disease Activity Index (DAI) scoring, colonic histopathology, inflammatory and antioxidant levels, and intestinal barrier function.

**Results and Discussion:**

SA-Q-CS MPs markedly enhanced the overall richness and diversity of the gut microbiota, enhancing the abundance of short-chain fatty acids (SCFAs)-producing bacteria, such as *Bacteroidales, Lactobacillales*, and *Lachnospiraceae*. These changes contributed to improved intestinal barrier function, better metabolic processes, and stronger defense mechanisms, thereby ameliorating UC induced by 3% dextran sulfate sodium (DSS) in C57BL/6J mice. Compared to the DSS group, the SA-Q-CS MPs treatment group showed significant improvements, with the Disease Activity Index (DAI) and histopathological scores reduced by more than 66.9%, pro-inflammatory factor levels decreased by 65%, antioxidant levels increased over sevenfold, and tight junction protein expression elevated by more than threefold. In conclusion, this investigation presents SA-Q-CS MPs as a promising strategy for restoring gut microbiome homeostasis and providing precise treatment for UC.

## Graphical abstract

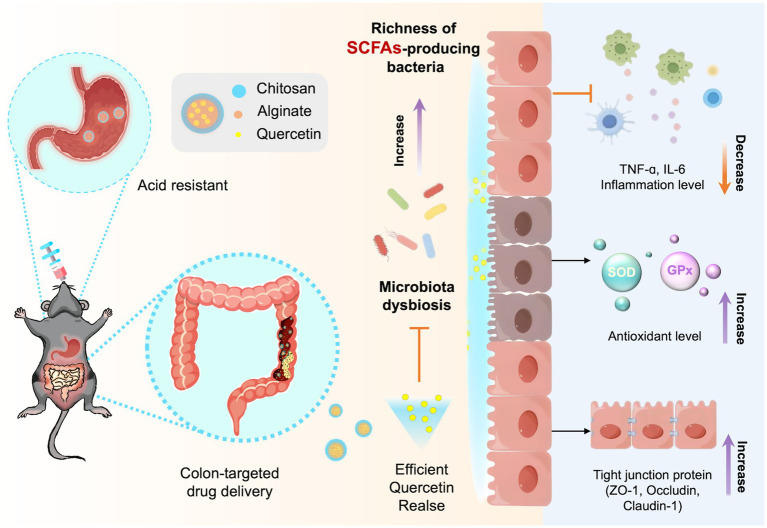

## 1 Introduction

Ulcerative colitis (UC) is a nonspecific intestinal disorder characterized by abnormal immune responses and intense inflammation (Berre et al., 2023; Fries et al., 2023). Evidence suggests that UC is closely linked to gut dysbiosis and intestinal barrier dysfunction (Lee et al., 2020). UC is difficult to cure and significantly impacts patients’ quality of life (QoL) (Sharara et al., 2018). Current UC treatments, including 5-aminosalicylic acid (5-ASA), corticosteroids, and biologics like infliximab and adalimumab, have limited impact on underlying causes such as intestinal barrier disruption and microbiota dysbiosis (Ni et al., 2017). Long-term immunosuppressant use can also cause serious complications, including immune dysregulation, opportunistic infections, and liver toxicity, highlighting the urgent need for innovative therapies to address the treatment challenges of UC (Zhao et al., 2021a; Ramos et al., 2023).

Short-chain fatty acids (SCFAs) are primary end-products generated by the colon’s microbiota through the fermentation of dietary fibers and play a crucial role in providing nutrients and energy to the host (den Besten et al., 2013; Koh et al., 2016; Fusco et al., 2023). They have been shown to modulate cell proliferation and function, exert anti-inflammatory and antibacterial effects, enhance intestinal integrity, and alleviate colitis (Shin et al., 2023; Ventura et al., 2024). In patients with colitis, the abundance of SCFAs is often significantly reduced (Mann et al., 2024). Supplementing SCFAs through exogenous routes has effectively reduced inflammation and intestinal damage in colitis.

Quercetin (QT) is a natural flavonoid compound widely found in plants. Research shows that quercetin effectively scavenges free radicals, reduces oxidative stress, and exhibits significant anti-inflammatory properties (Yang et al., 2018; Li et al., 2021). It can also improve gut microbiota composition by increasing the abundance of probiotic bacteria, such as SCFAs-producing bacteria, and promoting intestinal homeostasis (Zhao et al., 2021b; Hu et al., 2023; Liu et al., 2024). However, its poor water solubility and mucosal irritation hinder its oral application (Alizadeh et al., 2023; Zhuang et al., 2023). It has been reported that modifying drugs with biomacromolecules can enhance the bioavailability and biological activity of quercetin (Sun et al., 2020). Nevertheless, overcoming the acid environment in the stomach remains a significant challenge for oral medications (Hlaing et al., 2022; Heikal et al., 2023).

Sodium alginate (SA) and chitosan (CS) are promising biopolymer encapsulation carriers with excellent biocompatibility, biodegradability, low cost, and ease of processing, approved by the U.S. Food and Drug Administration (FDA) for food and drug applications (Nalini et al., 2019; Fernando et al., 2020). By adjusting the ratio and cross-linking degree of sodium alginate and chitosan, a drug delivery carrier with specific release properties in different pH environments can be designed, achieving precise control over drug release (Nalini et al., 2019; Chit et al., 2022). In acidic environments, it contracts to protect encapsulated drugs from gastric acid and proteolytic degradation, while in alkaline environments, it expands to facilitate drug release upon reaching the colon, demonstrating its colon-targeted drug delivery function (Takka and Gürel, 2010; Moreno-Rivas et al., 2024; Wang et al., 2025).

To address the challenges associated with ulcerative colitis (UC) treatment and enhance the colonic microenvironment, we developed colon-targeted sodium alginate-quercetin-chitosan microspheres (SA-Q-CS MPs) as a potential therapeutic strategy. We propose that SA-Q-CS MPs can specifically target colonic release, enhance short-chain fatty acid (SCFA)-producing bacteria, and thus alleviate DSS-induced ulcerative colitis. We explored the stability and pH responsiveness of SA-Q-CS MPs, confirming their resistance to gastric acid and ability to release quercetin in the colon selectively. The targeted delivery of quercetin significantly increased the abundance of short-chain fatty acid-producing bacteria, improved the anti-inflammatory and antioxidant properties of colonic epithelial cells, and enhanced the integrity of the intestinal barrier, offering a reference basis for the clinical management of UC.

## 2 Materials and methods

### 2.1 Materials

The materials used in this study included sodium alginate (SA, AR, molecular weight = 216.1 kDa, G/M = 1: 1, viscosity was 550 cP) was obtained from Macklin Biochemical Technology Co., Ltd. (Shanghai, China), chitosan (CS, BR, molecular weight = 161.2 kDa, degree of deacetylation>90%), glacial acetic acid (AR, molecular weight = 60.05 Da), anhydrous calcium chloride (CaCl_2_, AR, molecular weight = 110.98 Da), liquid paraffin (AR, molecular weight = 368.38 Da), anhydrous ethanol (AR, molecular weight = 46.07 Da), and Span80 (AR, molecular weight = 428.60 Da) were purchased from Sinopharm Chemical Reagent Co., Ltd. (Beijing, China). Petroleum ether (Beijing Chemical Industry Group Co., Ltd., Beijing, China) All chemical reagents used are analytical grade.

### 2.2 Preparation and characterization of sodium alginate-quercetin-chitosan microspheres

Microspheres of SA-Q-CS were produced using a modified emulsion crosslinking technique, incorporating CaCl_2_ as the crosslinking agent. The water phase contained SA and CS; the oil phase consisted of liquid paraffin and nonionic surfactant Span-80. To maximize quercetin encapsulation, the optimal ratio of materials was determined using factorial design, as shown in Supplementary Table S1. The swollen sodium alginate solution was mixed with quercetin powder at a mass ratio of 10:1. A total of 25 mL of the mixture was added slowly to the oil phase and was stirred and emulsified using magnetic stirrers (IKA, Germany) at 500 rpm for 30 min to form a water-in-oil (W/O) emulsion. Subsequently, 25 mL of CaCl_2_ solution was slowly added to the swollen chitosan solution, mixed evenly, and then added to the emulsion, stirring continuously for 4 h at 500 rpm to solidify the microspheres. The reaction mixture was then centrifuged to collect the SA-Q-CS MPs, which were purified 3 times each with petroleum ether, anhydrous ethanol, and distilled water, respectively. The final SA-Q-CS MPs were obtained by centrifugation at 10,000 rpm for 5 min and drying at low temperatures.

### 2.3 Characterization of SA-Q-CS MPs

Scanning electron microscopy (SEM, ZEISS Gemini 300, Germany) and transmission electron microscopy (TEM, JEM-F200, JEOL, Japan) were utilized to assess the physical properties of SA-Q-CS MPs. The surface voltage of SA-Q-CS microspheres was monitored by a Zeta potentiometer (Malvern, United Kingdom).

The encapsulation efficiency and drug loading of quercetin were determined indirectly. A standard curve for quercetin concentration was established based on absorbance measurements at 340 nm. Sodium tripolyphosphate (STPP) was used to depolymerize SA-Q-CS microspheres, and the quercetin concentration in the supernatant was measured to calculate encapsulation efficiency and drug loading. The entrapment efficiency (Equation 1; Nsereko and Amiji, 2002) and loading efficiency (Equation 2; Wang et al., 2004) of quercetin were calculated using established formulas.


(1) $$\%\text{Entrapment efficiency}=\frac{\text{Practical content}}{\text{Theoretical content}}\times 100$$

(2) $$\%\text{Loading efficiency}=\frac{\text{Practical content}}{\text{Microsphere quality}}\times 100$$

### 2.4 Release efficiency and stability of SA-Q-CS MPs *in vitro*

SA-Q-CS microspheres were exposed to simulated gastric fluid (SGF, pH 1.5), distilled water (pH 6.5), phosphate buffer (PBS, pH 7.0), simulated small intestine fluid (SIF, pH 7.6), and simulated colonic fluid (SCF, pH 8.3) (Coolaber Technology Co., LTD, China). The samples were incubated at 37°C on a uniform temperature shaking table (Zhichu Instrument Co., LTD., China). Supernatants were collected at various time points (10 min, 30 min, 1 h, 2 h, 3 h, 4 h, 5 h) and analyzed using a microplate reader (BioRad, iMark, United States) at 340 nm to determine quercetin concentration, calculated via a standard curve. Each measurement was performed in triplicate. SEM was utilized to analyze the morphology of MPs after 2 h of exposure to different pH solutions, in order to observe the release characteristics of quercetin under various pH conditions.

### 2.5 Experimental design of colitis modeling animal

A total of 36 female C57BL/6 J mice (6-week-old, obtained from Charles River Laboratories, China). Dextran Sulfate Sodium (DSS) was obtained from YEASEN Biotechnology Co., Ltd., FITC-labeled Dextran (MW 4000), as well as assays for total SOD activity (WST-8 method), glutathione peroxidase, Mouse TNF-*α* ELISA Kit, and Mouse IL-6 ELISA Kit, were procured from Beyotime Biotechnology Co., Ltd.

All animal experiments described in this study were conducted in accordance with the ARRIVE (Animal Research: Reporting of *In Vivo* Experiments) guidelines in accordance with Guidance on the operation of the Animals (Scientific Procedures) Act 1986, and with approval from the Animal Ethics Committee of China Agricultural University (Approval No AW02013202-2-2), mice were acclimatized for 1 week before being divided into six groups: healthy control, (DSS), quercetin treatment group (DSS+QT), SA-CS treatment group (DSS+SA-CS), sodium alginate-chitosan coated quercetin (DSS+SA-Q-CS) treatment, and 5-ASA drug sulphasalazine (SSZ) treatment groups (DSS+SSZ). Based on power calculation (effect size = 1.5, α = 0.05, power = 0.8) and 3R principles, to minimize animal use while maintaining scientific integrity, we selected a group size of *n* = 6. Mice in the control group received distilled water, while others drank water containing 3% sodium dextran sulfate for 7 days, followed by distilled water for 5 days of recovery. Daily records included body weight, fur condition, activity levels, stool consistency, and behavior.

On days 1, 3, 5, 7, 9, and 11 of the experiment, the control group received distilled water, while the other groups were administered distilled water, quercetin, SA-CS microspheres, SA-Q-CS microspheres, and SSZ solution (all at 50 mg/kg) orally. On day 12, mice were anesthetized by intraperitoneal injection of Zoletil® 100 (tiletamine-zolazepam, Virbac, France) at a dose of 50 mg/kg body weight. The depth of anesthesia was assessed by the absence of corneal and pedal withdrawal reflexes. Once deep anesthesia was confirmed, liver vein blood was collected, followed by euthanasia by cervical dislocation. The colons of different groups were excised and measured. Sections of colon tissue were fixed in 10% formaldehyde for histopathological analysis, while another part was frozen at −80°C.

### 2.6 Histopathological score of colons

Mice colon tissue slices were prepared following the standard procedures followed by staining with hematoxylin and eosin (H.E.), and the histological morphology of the colon was observed using a microscope (Nikon Y-TV55, Japan).

### 2.7 Serum levels of IL-6 and TNF-*α* in C57BL/6 J mice

Blood samples were collected into centrifuge tubes and left at room temperature for 2 h. They were then centrifuged at 3000 rpm at 4°C for 10 min to obtain serum, which was used for ELISA analysis of TNF-α and IL-6 levels using commercialized test kits (Beyotime Biotechnology, China). The absorbance was measured at 450 nm by a microplate reader (BioRad, iMark, United States). Each sample group was analyzed in triplicate.

### 2.8 Measurement of intestinal antioxidant capacity

Levels of antioxidant enzymes, including superoxide dismutase (SOD) and glutathione peroxidase (GPx), were measured in each group using commercial test kits (Beyotime Biotechnology, China). Fresh mouse colon samples were taken or frozen at −80°C (thawed at 4°C for 12 h before use). SOD and GPx were extracted from colon tissues following kit instructions. Each sample group was analyzed in triplicate.

### 2.9 Evaluation of intestinal mucosal barrier permeability

Three mice were randomly selected from each group following the treatment regimen. After completing the administration protocol, mice were orally administered 0.5 g/kg FITC-Dextran (Beyotime Biotechnology, China). Four hours later, mice were anesthetized with isoflurane, and blood was collected from the portal vein into heparin sodium anticoagulant tubes, which were kept on ice in the dark. Whole blood samples were transferred to a 96-well plate (200 μL per well), and fluorescence intensity of portal vein blood was measured at excitation wavelength 490 nm and emission wavelength 520 nm using a microplate reader to assess intestinal permeability.

### 2.10 Immunohistochemistry

Immunohistochemical (IHC) staining was used to detect colonic tight junction protein ZO-1. Colon tissue sections were dewaxed to water, subjected to antigen retrieval, blocked with 3% H_2_O_2,_ and incubated with primary antibodies. A 1:100 dilution of ZO-1 antibody was applied to slides and incubated at 4°C for 24 h. DAB color development and hematoxylin counterstaining were performed for visualization. Image J 2.14.0 software was utilized to analyze the gray value of positive signals; three different IHC images were selected from each group for analysis.

### 2.11 Western blotting

Colon tissue proteins from mice were extracted using RIPA buffer with PMSF, and protein levels were evaluated by the BCA assay kit (Beijing Solarbio Science and Technology Co., Ltd., Beijing, China). The proteins were separated by the 4–20% SDS-PAGE and transferred to a PVDF membrane (Merck KGaA, Darmstadt, Germany). The membranes were blocked with 5% skim milk for 1 h, then incubated overnight at 4°C with the primary antibodies: Claudin-1 (1:1,000, Boster Biological Technology Co. Ltd., Wuhan, China), Occludin (1:1,000, Proteintech Group, Inc., Wuhan, China), ZO-1 (1:1,000, Proteintech Group, Inc., Wuhan, China), and *β*-actin (1:1,000, Proteintech Group, Inc., Wuhan, China). The membranes were washed three times with TBST and then incubated with horseradish peroxidase-conjugated goat anti-mouse IgG or goat anti-rabbit IgG (Beyotime Biotechnology Co. Ltd., Shanghai, China) for 1 h. Target strips were visualized by chemiluminescence image analyzer (Tanon Science and Technology Co., Ltd., Shanghai, China).

### 2.12 Microbiota analysis

After euthanizing the mice, the colon contents were carefully collected and placed into a sterile centrifuge tube. The samples were then immediately frozen using liquid nitrogen. Total genomic DNA was extracted from colon content samples using the TGuide S96 Magnetic Stool DNA Kit (Tiangen Biotech, Beijing, China) following the manufacturer’s guidelines. The hypervariable region V3-V4 of the bacterial 16S rRNA gene was augmented by polymerase chain reaction (PCR), and PCR products were analyzed on an agarose gel and purified using the Omega DNA Purification Kit (Omega Inc., Norcross, GA, United States). The purified products were then collected, and paired-end sequencing (2 × 250 bp) was performed on the Illumina Novaseq 6000 platform. Sequences with similarity > 97% were clustered into the same operational taxonomic unit (OTU) by USEARCH (v10) (Edgar, 2013). Taxonomic annotation of the representative sequences was performed using a Naive Bayes classifier trained on the SILVA 138 reference database. Classification was conducted at multiple taxonomic levels (phylum, class, order, family, genus, and species) based on sequence similarity and classifier confidence scores. Differences in bacterial community composition and functional gene predictions were analyzed using QIIME2 2020.6 and PICRUSt2 software.

### 2.13 Statistical analysis

Data analysis was conducted using GraphPad Prism 10.0 (CA, United States). Results were expressed as the mean ± SD. All experiments were independently repeated three times. One-way ANOVA was used for comparing variables between groups. Statistical significance levels were defined as follows: ^*^p < 0.05; ^**^p < 0.01; ^***^p < 0.001; ^****^p < 0.0001; ns (not significant) *p* > 0.05.

## 3 Results

### 3.1 Synthesis and characterization of sodium alginate-quercetin-chitosan microspheres

The characterization of sodium alginate-quercetin-chitosan microspheres (SA-Q-CS MPs, including zeta potential, particle size, encapsulation rate, and drug loading efficiency), was assessed. As shown in Supplementary Table S1, increasing the concentration of SA caused the particle size of SA-Q-CS MPs to initially decrease and then increase, while the zeta potential exhibited the opposite trend. Increasing the ratio of chitosan to sodium alginate led to increases in particle size, encapsulation rate, and drug loading efficiency. However, the concentration of SA had a more significant impact on particle size than the crosslinking agent CaCl_2_, which did not notably affect particle size or drug load encapsulation rate. Microspheres with smaller particle sizes exhibited a larger specific surface area and higher encapsulation rate for quercetin, minimizing drug loss during the preparation process. This indicates that the polymer content is a crucial factor affecting the particle size of the microspheres.

**Figure 1:**
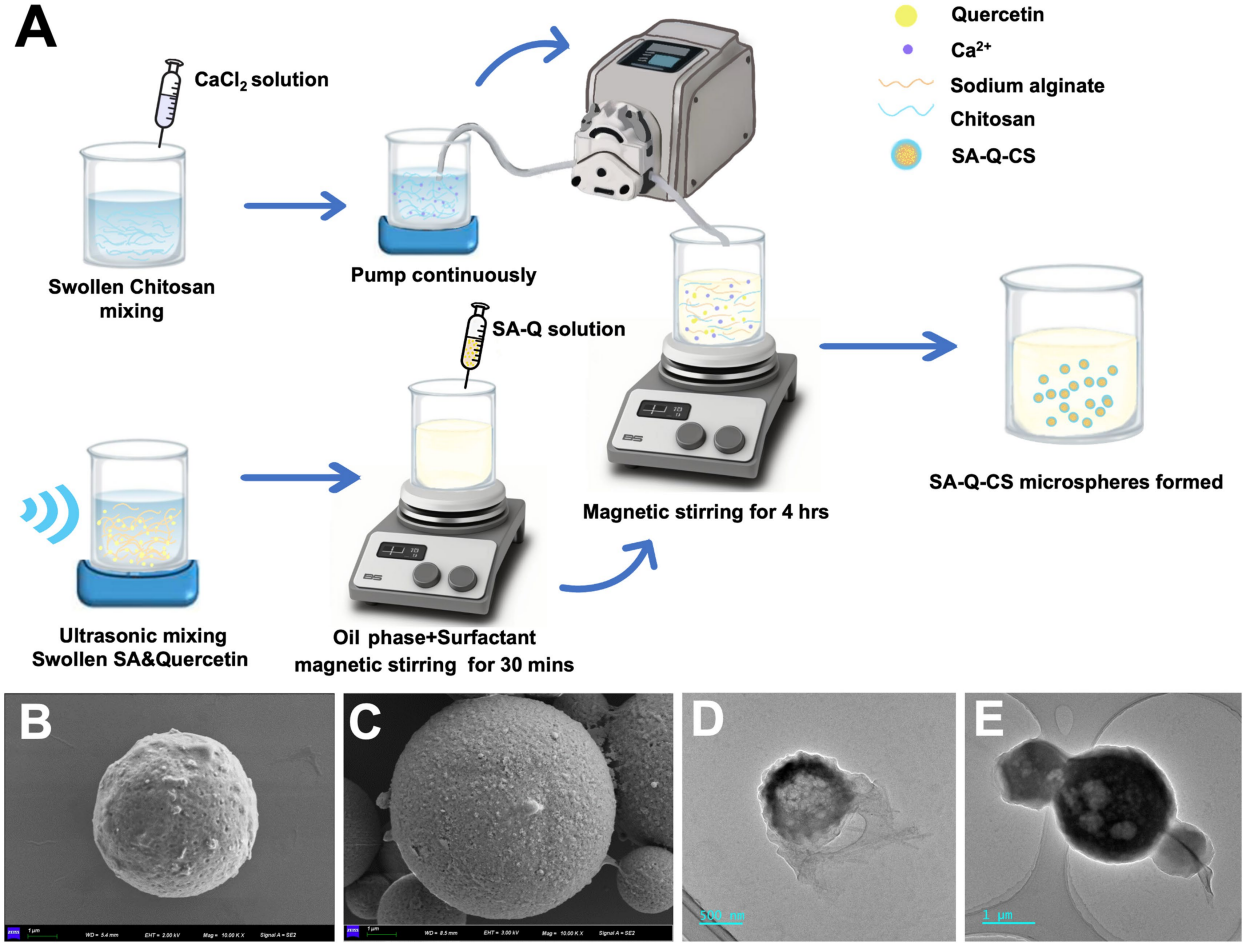
Preparation and characterization of materials. **(A)** Schematic outline of preparation of SA-Q-CS; **(B,C)** SEM image of CA MPs and SA-Q-CS MPs, scale bar = 1 μm; **(D)** TEM image of CA MPs, scale bar = 500 nm; **(E)** TEM image of SA-Q-CS MPs, Scale bar = 1 μm.

Based on the analysis results detailed in Supplementary Table S1, we optimized the encapsulation of quercetin using specific conditions: 1% (w/v) SA, 1% (w/v) CS, 25% (w/v) CaCl_2_, and a 10:1 mass ratio of SA to quercetin. The material preparation process is outlined in Figure 1A. We employed an emulsion crosslinking method to prepare SA-CS microspheres and SA-Q-CS MPs. The drug loading and encapsulation efficiency were measured through an indirect process. The average particle size of SA-Q-CS MPs was determined at 3.25 ± 0.26 μm. The encapsulation efficiency was 81.20%, while the drug loading efficiency was 15.1%. The Zeta potential of the SA-Q-CS microspheres was −41.3 ± 0.59 mV.

The SEM image of the SA-Q-CS MPs and the unloaded SA-CS MPs are depicted in Figures 1B,C. Both types of microspheres exhibit spherical shapes with rough surfaces and uniform morphology. TEM images showed that SA-Q-CS MPs exhibited higher electron density inside, and the particle size (Figure 1D) was larger than that of SA-CS MPs (Figure 1E).

### 3.2 *In vitro* drug release efficiency and stability test

**Figure 2:**
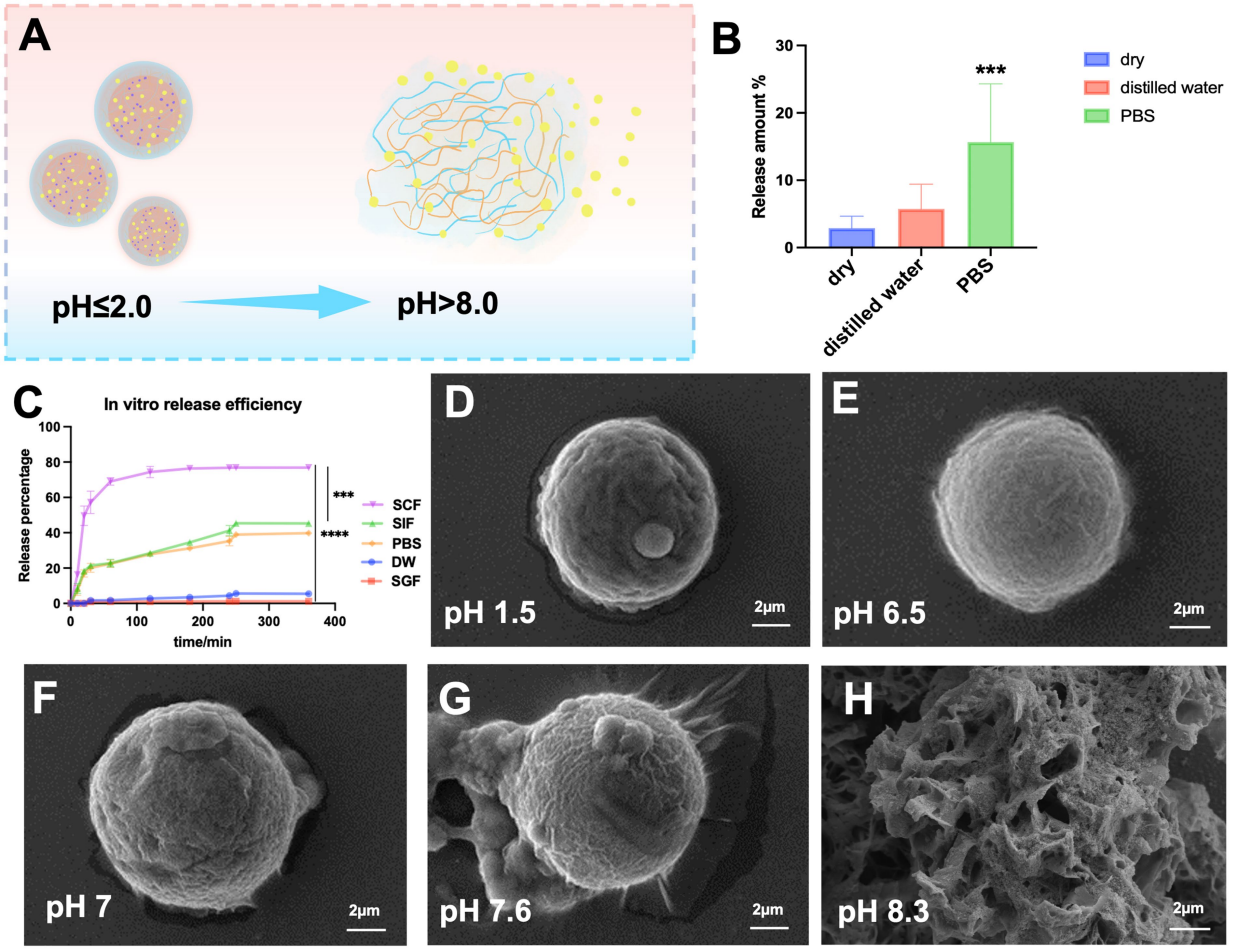
Drug release efficiency and stability of SA-Q-CS MPs *in vitro*. **(A)** Schematic diagram of SA-Q-CS MPs at different pH; **(B)** Stability test of SA-Q-CS MPs in different environments; **(C)** Release efficiency curve in vitro; **(D–H)** SEM images of the morphology of SA-Q-CS MPs in various pH fluids, **(D)** simulated gastric fluid (SGF), **(E)** distilled water (DW), **(F)** phosphate buffer (PBS), **(G)** simulated small intestine fluid (SIF), **(H)** simulated colonic fluid (SCF). Data was represented as mean ± SD (^**^p < 0.01, ^***^p < 0.001 by one-way ANOVA with Dunnett’s multiple comparisons test).

To assess the stability and pH response of SA-Q-CS MPs, *in vitro* evaluations of their release efficiency and stability were evaluated. Figure 2A presents the drug release diagram of SA-Q-CS MPs. In an acidic environment, the protonation of MP molecules reduces the surface negative charge and intermolecular electrostatic repulsion, causing the molecules to contract. The contraction protects the internal drug from stomach acid and protease hydrolysis. Conversely, in an alkaline environment, SA-Q-CS MPs deprotonate, causing the molecules to disperse and swell, transitioning from a dense to a loose structure and releasing the encapsulated quercetin.

Figure 2B shows the stability evaluation of SA-Q-CS MPs in vitro. After 7 days in a dry environment and distilled water, drug release was less than 5%. However, after 7 days in PBS, quercetin release significantly increased to 18%, indicating that SA-Q-CS MPs are stable in dry environments and distilled water but release quercetin more readily in PBS (*p* < 0.001).

To evaluate the pH response capability of SA-Q-CS MPs, their release efficiency in simulated digestive solutions, PBS, and distilled water at different pH values were tested. Figure 2C displays the in vitro release efficiency results. In SGF and distilled water, the final quercetin release efficiency was below 5%. In SIF and PBS, an initial release plateau was observed at 60 min, followed by a second plateau at 240 min, with a final release efficiency of approximately 40%. The release plateau occurred at 60 min, reaching a significantly higher final release efficiency of 80%. The release efficiency in SCF was significantly higher than in other lower pH environments (*p* < 0.001).

SEM images (Figures 2D–H) show the surface morphology of SA-Q-CS microspheres after 2 h in different media. The microspheres remained intact in SGF (Figure 2D) and distilled water (Figure 2E). In contrast, partial surface depolymerization was evident in SIF (Figure 2F) and PBS (Figure 2G). In SCF (Figure 2H), the structure transformed into a loose, amorphous form. These results indicated that SA-Q-CS MPs had excellent pH responsiveness, effectively protecting the encapsulated quercetin from gastric acid and other digestive fluid hydrolysis, and had the potential for colon-targeted drug delivery.

### 3.3 Oral SA-Q-CS MPs alleviate the symptoms of DSS-induced colitis disease in mice

**Figure 3:**
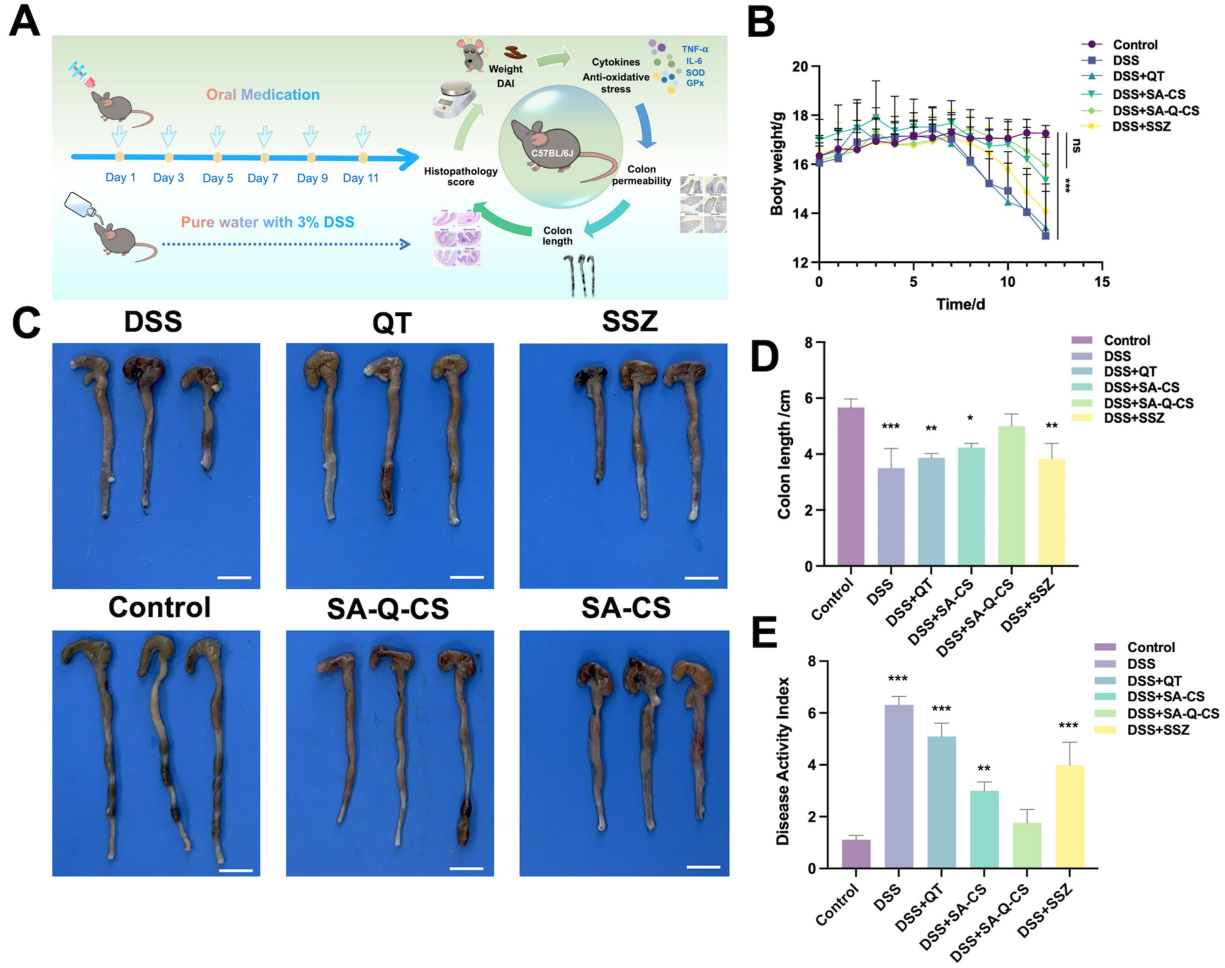
Oral administration of SA-Q-CS MPs improves clinical symptoms in mice with DSS-induced colitis. **(A)** C57BL/6 J mice colitis modeling and treatment plan and drug efficacy test indexes, the oral dose of each group was 50 mg/kg BW/ time. **(B)** Body weight loss of C57BL/6 J mice. **(C)** Colonic images of mice in each group, scale bar 1 cm; **(D)** Colon length of mice in each group; **(E)** Disease activity index (DAI) score. Data was represented as mean ± SD (ns, *p* > 0.05; ^*^p < 0.05, ^**^p < 0.01, ^***^p < 0.001, and ^****^p < 0.0001 by one-way ANOVA with Dunnett’s multiple comparisons test, *n* ≥ 3).

The *in vivo* colitis treatment experimental scheme is illustrated in Figure 3A. When the experiment was completed, the weight of mice in the DSS model group decreased by 20% (Figure 3B). Following treatment with various drugs, the model mice showed an increase in weight. Notably, the DSS+SA-Q-CS group exhibited weight recovery comparable to the control group (*p* > 0.05). Colon images and colon length data for all groups are shown in Figures 3C,D. After six doses of treatment, the colon length of the SA-Q-CS group increased by 30.4% compared to the DSS group. The result indicated that SA-Q-CS can effectively alleviate the colon shortening typically caused by DSS. In contrast with the control group, the colon length in the other treatment groups was shortened to various degrees, with the degree of shortening as follows: QT treatment group (*p* < 0.01) > SSZ treatment group (*p* < 0.01) > SA-CS treatment group (*p* < 0.05).

The Disease Activity Index (DAI) score was calculated based on three parameters: body weight loss, stool consistency, and fecal occult or gross blood. Higher DAI scores indicate more severe colitis symptoms. The DAI scores presented in Figure 3E showed that the score of the SA-Q-CS group decreased by 66.7% compared to the DSS group. While SA-CS also significantly alleviated the increase in DSS-induced DAI scores, it did not fully restore them to healthy levels. The remission of DAI after 5-ASA drugs, SSZ, and QT was limited. These results demonstrated that SA-Q-CS treatment outperformed other treatment strategies, indicating that oral SA-Q-CS MPs can effectively alleviate the colitis symptoms in mice.

### 3.4 Oral SA-Q-CS MPs improve both colon inflammation and antioxidant levels

**Figure 4:**
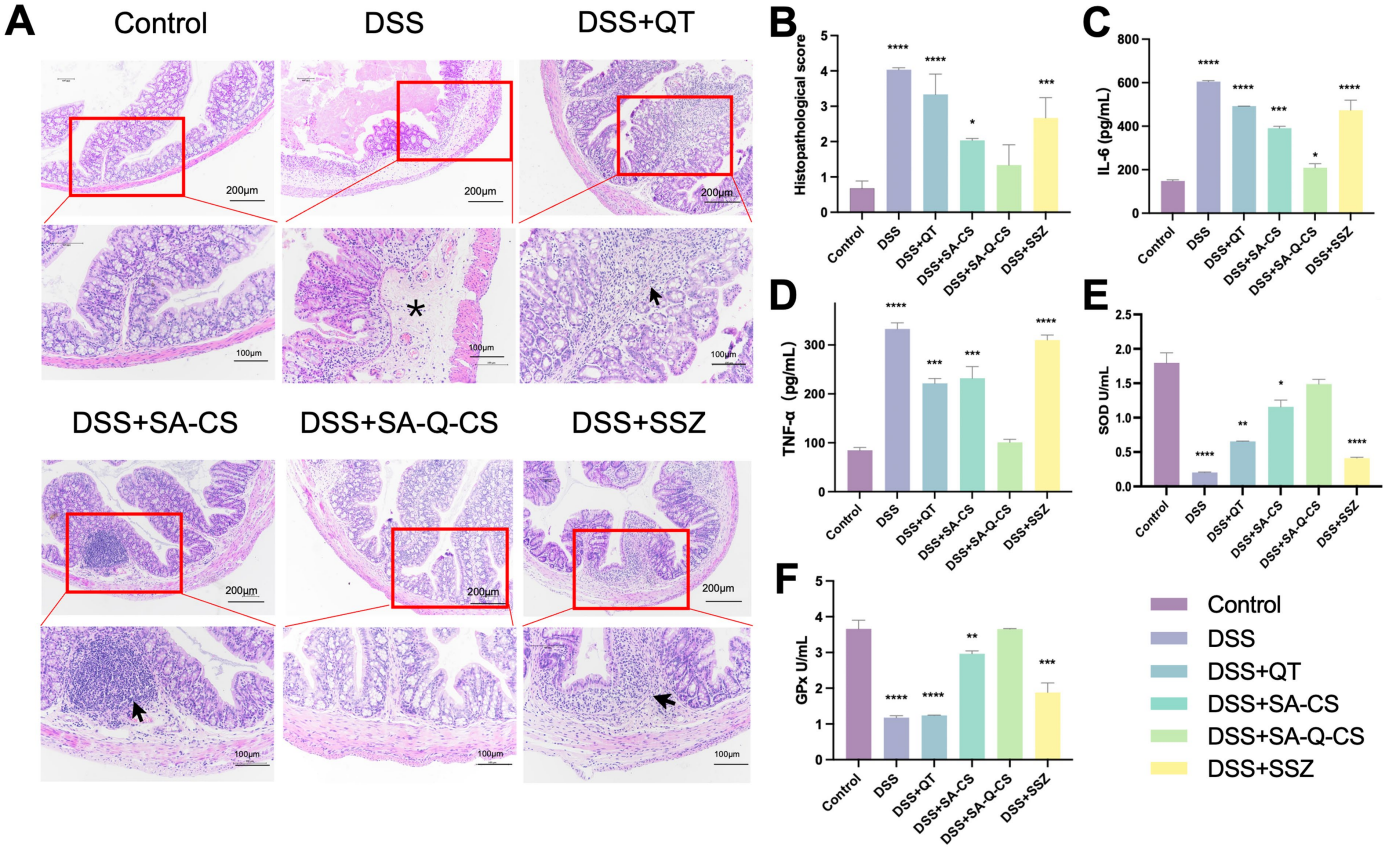
Oral administration of SA-Q-CS MPs ameliorates colon histopathology, serum pro-inflammatory cytokine levels, and antioxidant function. **(A)** H.E. staining images with low power (scale bar 200 μm) and high power (scale bar 100 μm), black arrows indicate proliferation and infiltration of inflammatory cells, and asterisk (*) indicates vascular congestion; **(B)** Histopathological score; **(C)** Serum IL-6; **(D)** Serum TNF-α level. **(E)** Detection of SOD activity in intestinal tissue of mice; **(F)** Detection of GPx activity in mouse intestinal tissue. Data was represented as mean ± SD (^*^p < 0.05, ^**^p < 0.01, ^***^p < 0.001, and ^****^p < 0.0001 by one-way ANOVA with Dunnett’s multiple comparisons test, *n* ≥ 3).

Histopathological analysis of the colon in each group was shown in Figure 4A. H.E. staining sections in the DSS model group exhibited severe inflammatory cell infiltration, blood vessel congestion, extensive mucosal damage, and crypt disappearance compared to the control group. Treatment with QT, SA-CS, SA-Q-CS, and SSZ improved the colitis to varying degrees. Among these groups, SA-Q-CS treatment resulted in the most significant remission of inflammation, with reduced inflammatory cell infiltration, improved crypt integrity, and minimized inflammatory area. The histopathological score of the SA-Q-CS group showed no significant difference compared to the control group (*p* > 0.05). The SA-CS treatment group also showed some recovery in histopathological morphology, but the histopathological score remained higher than that of the control group (*p* < 0.05). In contrast, the QT and SSZ treatment groups displayed more severe inflammatory cell infiltration and colonic wall damage, with significantly higher histopathological scores compared to the healthy control group (*p* < 0.001) (Figure 4B).

The expression levels of pro-inflammatory cytokines (IL-6 and TNF-*α*) in the serum of each group were analyzed using ELISA commercial kits, as depicted in Figures 4C,D. Following SA-Q-CS treatment, IL-6 levels in colitis mice decreased by 66.7%, markedly reduced its congregation. TNF-α levels decreased by 69.8%, approaching those of the control group (*p* > 0.05). The serum levels of IL-6 and TNF-α in the QT, SA-CS, and SSZ groups were notably elevated compared to healthy mice (*p* < 0.001).

To investigate the oxidation resistance in the colon, the superoxide dismutase (SOD) and glutathione peroxidase (GPx) levels of each group were also evaluated (Figures 4E,F). UC mice showed significantly reduced SOD and GPx levels, indicating lower antioxidant capacity and increased oxidative stress, which contributed to intestinal tissue damage. Following treatment, SOD and GPx levels in the SA-Q-CS group significantly increased, returning to healthy levels and bolstering antioxidant capacity. The SA-CS group also showed notable increases in SOD and GPx levels. In contrast, GPx levels in the QT and SSZ treatment groups resembled those of untreated UC mice, suggesting limited efficacy in improving intestinal antioxidant capacity. These results indicated that oral SA-Q-CS MPs alleviated the histological changes and cytokine release, strengthened antioxidant defenses, and protected against oxidative damage.

### 3.5 Oral SA-Q-CS MPs improve intestinal barrier function

**Figure 5:**
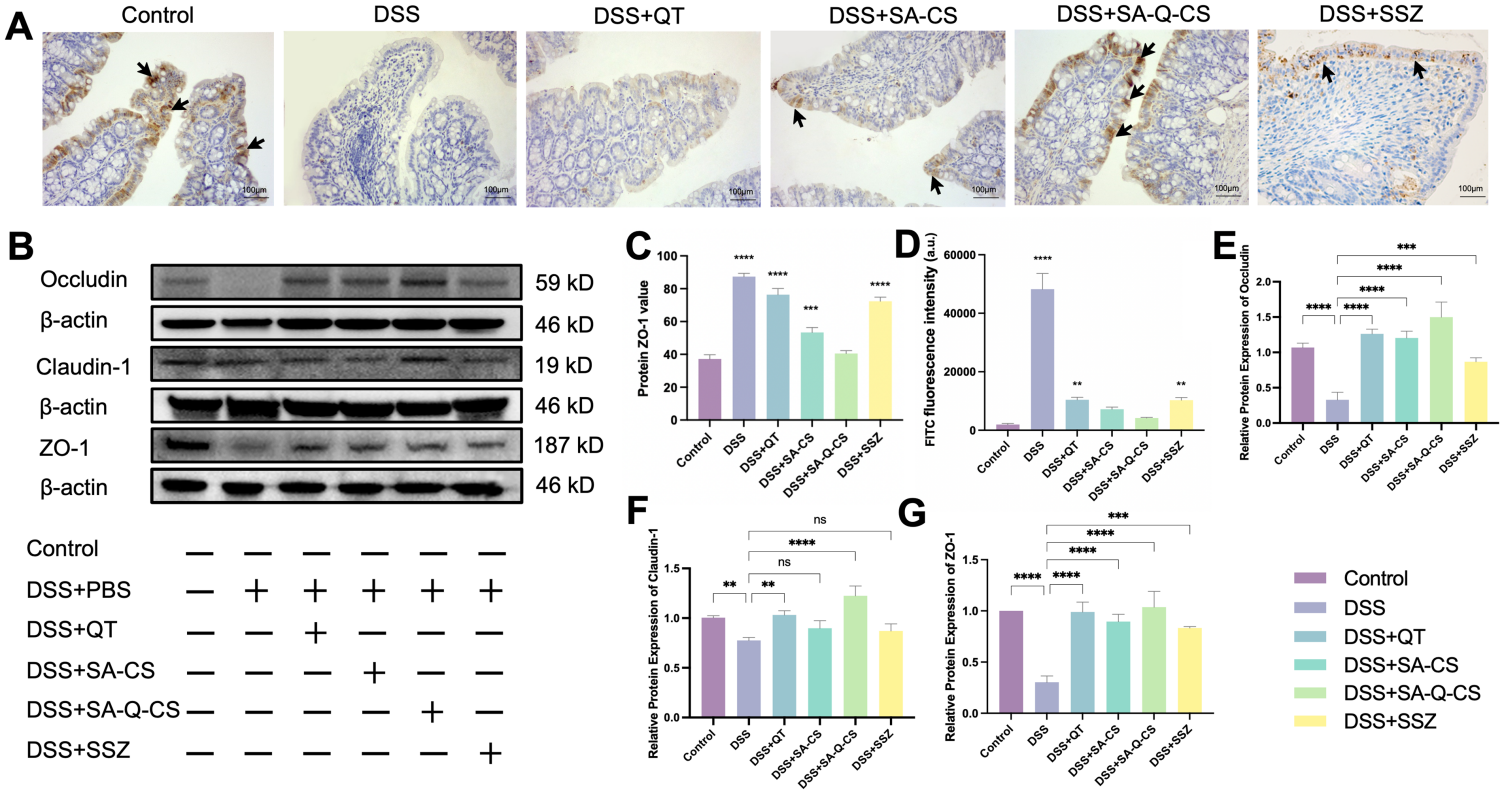
Oral administration of SA-Q-CS MPs improves the levels of colonic tight-junction proteins. **(A)** IHC staining images of ZO-1 protein, black arrows indicate positive signal of ZO-1 protein (scale bar 100 μm); **(B)** Protein bands of Occludin, Claudin-1 and ZO-1; **(C)** IOD was counted of ZO-1 by immunohistochemical staining; **(D)** FITC fluorescence intensity in blood of C57/BL6 mice in each FITC group; (**E–G**) Protein statistical analysis of proteins Occludin, Claudin-1, and ZO-1. Data was represented as mean ± SD (^*^p < 0.05, ^**^p < 0.01, ^***^p < 0.001 and ^****^p < 0.0001 by one-way ANOVA with Dunnett’s multiple comparisons test, *n* ≥ 3).

DSS-induced colitis decreased the expression of intestinal tight junction proteins such as Occludin, Claudin-1, and ZO-1 (Figures 5A,B). There is an inverse relationship between the grayscale values and the intensity of positive staining (Figure 5C). Compared to the healthy control group, the DSS-induced colitis group showed little positive signal in intestinal epithelial cells (*p* < 0.0001). SA-Q-CS MPs treatment group showed significantly increased ZO-1 positive signals.

The intensity of FITC fluorescence in blood was detected to assess colon permeability in different groups. As shown in Figure 5D, oral administration of SA-Q-CS MPs treatment reduced the blood FITC fluorescence intensity by 91.3% compared to the DSS group (*p* < 0.01), with no difference compared with the control group. Oral SA-CS MPs reduced fluorescence intensity by 85%, SSZ by 78.6%, and QT by 78.0%.

Figures 5E–G displays the Western blot analysis of colonic tight junction proteins. Compared to the colitis model group, all treatment groups showed varying degrees of increased protein expression. Specifically, the levels of Occludin and ZO-1 were considerably higher in all treatment groups than in the model group (*p* < 0.001). Both the SA-Q-CS MPs and QT treatment groups showed a significant increase in Claudin-1 expression compared to the model group (SA-Q-CS MPs: *p* < 0.0001, QT: *p* < 0.01). In contrast, no significant difference in Claudin-1 expression was found between the model group and either the SA-CS MPs or SSZ treatment groups.

These findings demonstrate that SA-Q-CS MPs effectively improve intestinal barrier function and reduce inflammation-induced intestinal permeability.

### 3.6 Oral SA-Q-CS MPs increase the richness of SCFAs-producing bacteria in the colon

**Figure 6:**
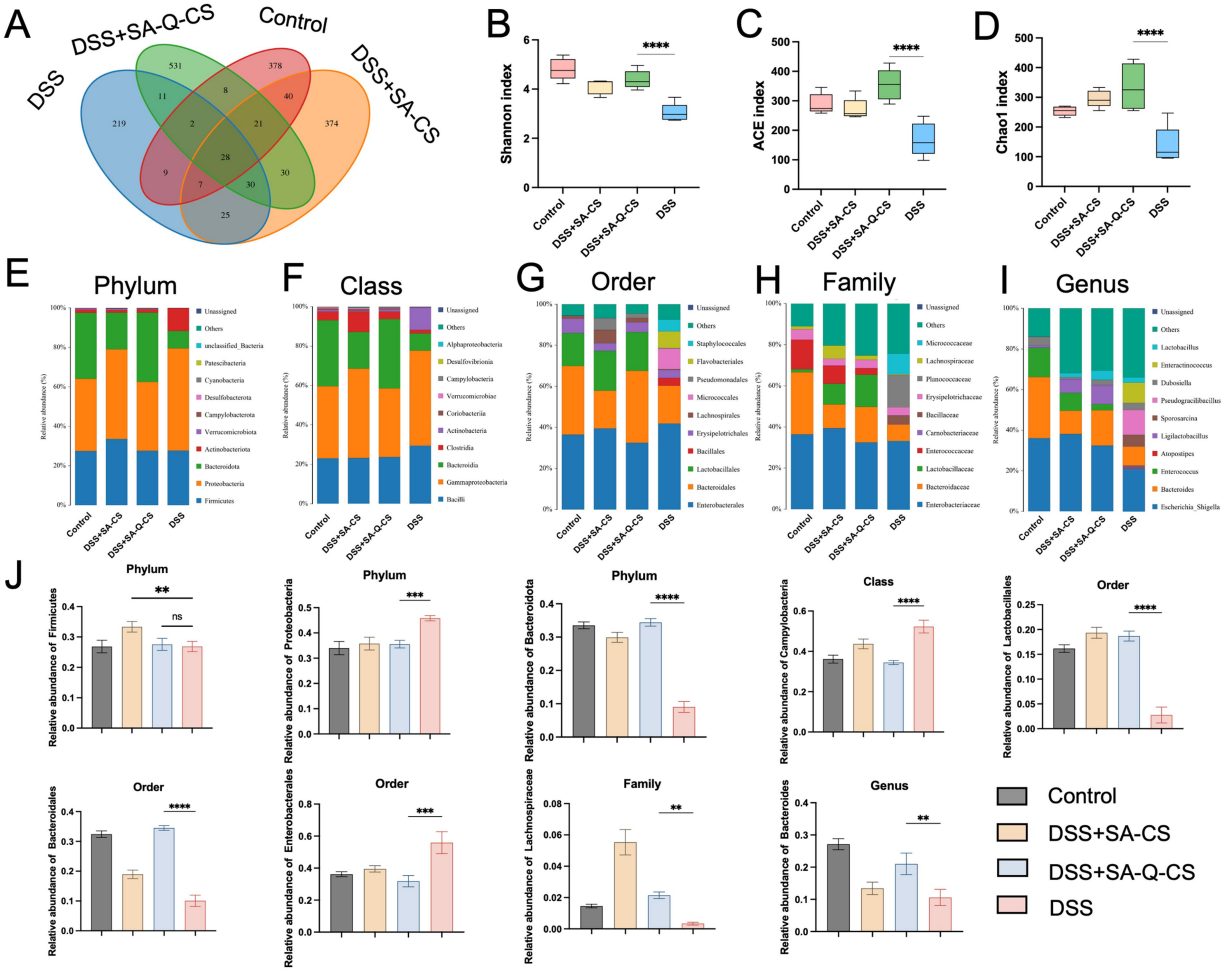
Oral administration of SA-Q-CS MPs changes the colon’s microbiota in DSS-treated mice. **(A)** Venn diagram of colonic flora composition in each group. **(B)** Shannon index. **(C)** ACE index. **(D)** Chao1 index. **(E–I)** Distribution of colon microbiota at the phylum, class, order, family, and genus levels, with the top 10 species displayed based on abundance. **(J)** Relative abundance of potential probiotics and pathogens in the colon in each group. Data was represented as mean ± SD (^**^p < 0.01, ^***^p < 0.001, and ^****^p < 0.0001 by one-way ANOVA with Dunnett’s multiple comparisons test, *n* ≥ 3).

High throughput 16S ribosomal RNA gene sequencing was performed on the colonic contents of animals from each group. Venn diagrams were used to display the Operational Taxonomic Units (OTUs) in each group, showing the highest similarity between the SA-Q-CS MPs treatment group and the healthy control group, while the model group exhibited the lowest similarity with the control group (Figure 6A). DSS-induced colitis resulted in a significant decrease in bacterial richness and diversity (Figures 6B–D). In contrast, SA-Q-CS treatment significantly improved the ACE, Chao1, and Shannon indices (*p* < 0.0001).

Furthermore, comprehensive taxonomic profiling of the colon microbiota was conducted at the phylum, class, order, family, and genus levels using a Naive Bayes classifier trained on the SILVA reference database. The resulting community composition across treatment groups was visualized to assess microbial structural differences. The relative abundance of species was represented by the length of colored blocks (Figures 6E–I). Analysis of the relative abundance at each taxonomic level revealed that the model group significantly altered the gut microbiota composition, decreasing the abundance of SCFAs-producing bacteria *Bacteroidota*, *Bacteroidales*, and *Lachnospiraceae* as well as probiotics like *Lactobacillales*, while increasing the abundance of pathogenic bacteria *Proteobacteria*, *Campylobacteria*, and *Enterobacterales* (Figure 6J). Treatment with SA-Q-CS MPs reversed these changes, significantly restoring the richness of SCFAs-producing bacteria. Specifically, the abundance of *Bacteroidales* increased by 3.4-fold, *Lachnospirales* by 6.4-fold, and the abundance of the probiotic *Lactobacillales* by 6.7-fold, thereby improving gut dysbiosis (*p* < 0.01).

### 3.7 Oral SA-Q-CS MPs improved the function of gut microbiota

**Figure 7:**
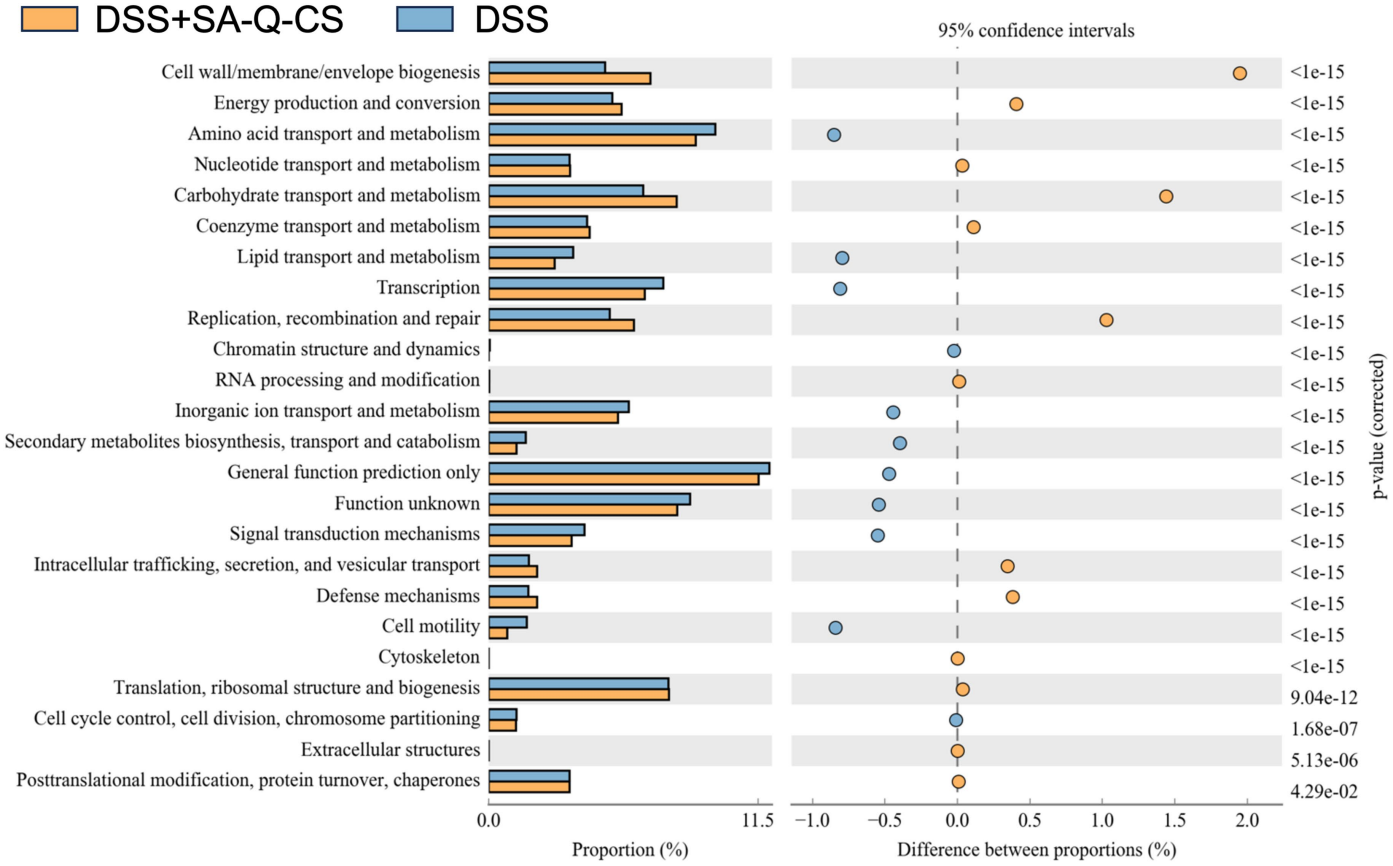
COG analysis of colonic microbiota in the SA-Q-CS MPs treated group vs. the untreated group. The functional distribution and abundance of microbiota sequences with significant differences (*p* < 0.05) are exhibited by COG categories.

The Clusters of Orthologous Groups of proteins (COG) functional classification of the intestinal microbiota in both the SA-Q-CS MPs treated and untreated enteritis groups is shown in Figure 7. All COG functional predictions were analyzed using a T-test, and those with significant differences (*p* < 0.05) were identified. Key differences were observed in the categories of cell wall*/*membrane*/*envelope biogenesis, carbohydrate transport and metabolism, replication, recombination and repair, intracellular trafficking and secretion, vesicular transport, and cytoskeleton functions.

Based on COG functional predictions, SA-Q-CS MPs treatment may potentially promote the proliferation and repair of intestinal epithelial cells by influencing pathways related to cell wall and membrane biosynthesis, as well as DNA replication and repair. These findings also suggest possible improvements in carbohydrate metabolism and short-chain fatty acid production, which may contribute to enhanced barrier function and reduced inflammation. Additionally, predicted enhancements in intracellular transport, secretion, and vesicle trafficking may be associated with improved cytoskeletal function, which could support the clearance of inflammatory mediators and aid in tissue remodeling.

## 4 Discussion

Due to the complex and multifactorial nature of UC pathogenesis, the development of effective clinical treatments has been limited (Salim and Söderholm, 2011; Gros and Kaplan, 2023). Recent studies have shown that the richness and diversity of the gut microbiota in UC patients have been significantly reduced, accompanied by a notable decline in short-chain fatty acid (SCFA)-producing bacteria. These changes disrupted colonic epithelial metabolism and permeability, leading to the accumulation of reactive oxygen species and triggering inflammatory responses in the colon (Sekirov et al., 2010; Nicholson et al., 2012; Koh et al., 2016). Here, we demonstrated the restorative effect of Quercetin-encapsulated with sodium alginate-chitosan microspheres (SA-Q-CS MPs) on intestinal microbiota and barrier dysfunction in DSS-induced UC mice. To tackle the problem of quercetin’s low solubility in the body, sodium alginate, and chitosan were used as the encapsulating agents through an enhanced emulsification cross-linking method (Raza et al., 2022; Chen et al., 2023; Li et al., 2025). The SA-Q-CS MPs demonstrated pH-responsive capability, enabling controlled drug release. In SGF, they maintained a dense structure with minimal release, while in SCF, the structure loosened, releasing over 80% of the drug within 2 h. Their strong resistance to gastric acid is a fundamental prerequisite for our subsequent research.

We further investigated the effect of SA-Q-CS MPs in DSS-induced UC models. Oral administration of SA-Q-CS improved weight loss, reduced DAI scores, and increased colon length in the UC mice, and the histopathological scores were reduced by more than 66.9%. Pro-inflammatory cytokines TNF-*α* (tumor necrosis factor-α) and IL-6 (interleukin-6) are critical indicators in UC (Iwakura and Ishigame, 2006; McCarthy et al., 2013; Wang et al., 2019; Ansari et al., 2021). The serological analysis showed that SA-Q-CS MP treatment decreased serum levels of TNF-α and IL-6 by more than 65% compared to the untreated group. Redox homeostasis is crucial for the overall balance of intestinal health (Zhu and Li, 2012; Dudzińska et al., 2018; Shi et al., 2022; Rodrigues Junior et al., 2023). Superoxide dismutase (SOD) and glutathione peroxidase (GPx) are essential antioxidant enzymes. Reflecting the antioxidant function of the body. Oral SA-Q-CS MPs can significantly increase SOD and GPx levels in mice by sevenfold. Intestinal tight junctions form the structural basis of the gut barrier, serving as the first line of defense against injury (Turner, 2009; Moonwiriyakit et al., 2023). ZO-1, a PDZ domain-containing protein, interacts with occludin and Claudin-1 to ensure the stability and proper functioning of tight junctions (Van Itallie et al., 2017; Cao et al., 2018; Su et al., 2024). DSS-induced UC typically results in a decrease in the content of tight junction protein (Hussain and Park, 2022). In this study, SA-Q-CS treatment restored the expression of tight junction proteins in UC mice, resulting in more than a threefold rise in the expression of these proteins in colon tissues, restoring them to healthy levels. These results suggest that SA-Q-CS can reduce inflammation, enhance antioxidant defense, and improve intestinal barrier integrity.

Short-chain fatty acids (SCFAs), including acetate, propionate, and butyrate, serve as the primary energy source for colonic cells and are essential for intestinal health, possessing anti-inflammatory properties by regulating G protein-coupled receptors (GPCRs), a type of immune cell receptors (Parada Venegas et al., 2019; Markowiak-Kopeć and Śliżewska, 2020; Hodgkinson et al., 2023). Additionally, they support barrier function by promoting mucus production and strengthening tight junctions between epithelial cells (Ma et al., 2022). Consistently, our study found the microbiota richness and diversity of UC mice were significantly reduced, with a marked decrease in SCFAs-producing bacteria such as *Bacteroidales*, *Lachnospiraceae,* and *Lactobacillales*. Conversely, harmful bacteria, including *Proteobacteria, Enterobacterales,* and *Campylobacterota*, were abundant in the colon. Overgrowth of these bacteria can trigger intestinal inflammation (Caenepeel et al., 2020). Treatment with SA-Q-CS MPs reversed the dysbiosis, improving gut microbiota composition, resulting in a 3.4-fold increase in *Bacteroidales* abundance, a 6.4-fold increase in *Lachnospirales*, and a 6.7-fold increase in *Lactobacillales.* The COG functional prediction analysis of the gut microbiota revealed that SA-Q-CS MPs significantly enhanced the abundance of OTUs related to *cell wall*/*membrane*/*envelope* biogenesis, *carbohydrate transport and metabolism*, and *replication, recombination*, *and repair.* Improving cell membrane and envelope integrity is directly related to gut barrier function (Liu et al., 2021; Xiong et al., 2022). Enhancing carbohydrate transport boosts SCFA production, regulates the immune environment, reduces inflammation, and inhibits harmful bacteria (Campos-Perez and Martinez-Lopez, 2021; Kaczmarczyk et al., 2022). Furthermore, significant improvements were observed in *intracellular trafficking, secretion, vesicular transport*, and the *cytoskeleton function,* both of which are crucial for maintaining intestinal barrier function (López-Posadas et al., 2017; Zhen et al., 2024). While these exploratory findings indicate possible mechanisms requiring future molecular validation. These results indicate that SA-Q-CS MPs improved gut barrier function by altering the gut microbiota, raising the richness of SCFA-producing bacteria, and thereby alleviating intestinal inflammation and barrier dysfunction. Given the scope of the present study and to minimize biological variability, only female mice were included. The absence of a blank SA-CS group is also noted. Future studies should include both sexes to better capture sex-related differences in therapeutic response and microbiota interactions and also incorporate a group receiving SA-CS without DSS exposure to clarify the carrier’s standalone effects.

Collectively, our research demonstrates that colon-targeting SA-Q-CS MPs offer excellent efficacy in UC treatment by enriching SCFA-producing bacteria. SA-Q-CS MPs can not only alleviate inflammation precisely but also improve intestinal barrier function. This work presents a promising strategy that addresses the underlying causes of UC.

## 5 Conclusion

In the current study, we successfully fabricated the pH-responsive SA-Q-CS MPs targeted drug delivery to the colon. These MPs significantly alleviated UC symptoms in mice, reduced inflammation, and restored antioxidant defenses in the colon. Additionally, SA-Q-CS MPs increased the tight junction protein expression and reduced intestinal permeability. Moreover, we further discovered that SA-Q-CS MPs improved the richness of gut SCFA-producing bacteria in UC mice, enhancing energy metabolism and barrier repair function in the microbiota. Overall, this approach shows great potential for UC treatment, with microbiota modulation and barrier function enhancement as promising therapeutic strategies.

## Data Availability

The raw data supporting the conclusions of this article will be made available by the authors, without undue reservation.
