# Supplementary material for: Enrichment of short-chain fatty acid-producing bacteria by pH-responsive sodium alginate and chitosan-encapsulated quercetin
Source: Front Microbiol. 2025 Aug 6;16:1594012. doi: 10.3389/fmicb.2025.1594012 (PMC12364867; doi:10.3389/fmicb.2025.1594012)
Supplement: Supplementary file 1 [file Table_1.DOCX]

Supplementary Material

Enrichment of Short-Chain Fatty Acid-Producing Bacteria by pH-Responsive Sodium Alginate and Chitosan-Encapsulated Quercetin

**Qianyu Bai^1, †^, Zhongling Zhao^2, †,^ Yujing Duan ^3, †^, Runqiu Cai^1^, Yinzhu Chen^1^, Chaoyu Zhou^1^, Xinyuan Tian^1^, Yifei Yang^1^, Haiyan, Wu^1^, Mingju Li^4^, Jia You^5^, Qingyi Song^6^, Hong Dong^2, *^ and Tianlong Liu^1, *^**

*** Correspondence:** Tianlong Liu: liutianlong@cau.edu.cn; Hong Dong: donghong@bua.edu.cn

† These authors contributed equally to this work.

## Supplementary Tables

Supplementary Table S1. Evaluation of SA-Q-CS microspheres.

| Batch code | Sodium alginate conc. (w/v%） | Chitosan conc.（w/v） | Crosslinking agent (CaCl_2_ conc. %W/V) | Zeta potential  （mV） | Particle Size（μm） | Entrapment efficiency（%） | loading efficiency（%） |
| --- | --- | --- | --- | --- | --- | --- | --- |
| 1-1-1 | 0.5 | 0.5 | 5 | -15.5±1.18 | 9.25±0.38 | 51.3% | 14.3% |
| 1-1-2 | 0.5 | 1 | 5 | -14.1±0.49 | 9.11±1.25 | 51.5% | 14.1% |
| 1-1-3 | 0.5 | 1.5 | 5 | -17.5±0.39 | 8.94±0.66 | 55.2% | 15.2% |
| 1-2-1 | 0.5 | 0.5 | 15 | -15.6±0.27 | 6.15±1.03 | 55.6% | 14.5% |
| 1-2-2 | 0.5 | 1 | 15 | -15.7±1.59 | 6.57±1.97 | 54.7% | 14.7% |
| 1-2-3 | 0.5 | 1.5 | 15 | -14.1±0.22 | 6.23±0.89 | 55.5% | 14.3% |
| 1-3-1 | 0.5 | 0.5 | 25 | -17.1±1.59 | 7.05±0.98 | 58.4% | 15.3% |
| 1-3-2 | 0.5 | 1 | 25 | -15.6±0.47 | 7.24±0.41 | 58.6% | 15.2% |
| 1-3-3 | 0.5 | 1.5 | 25 | -17.5±1.19 | 6.68±0.53 | 59.0% | 15.3% |
| 2-1-1 | 1 | 0.5 | 5 | -18.6±0.67 | 8.17±0.66 | 62.5% | 14.9% |
| 2-1-2 | 1 | 1 | 5 | -19.9±0.61 | 8.06±0.52 | 61.3% | 14.8% |
| 2-1-3 | 1 | 1.5 | 5 | -17.9±0.79 | 8.53±1.59 | 62.1% | 14.6% |
| 2-2-1 | 1 | 0.5 | 15 | -15.5±0.27 | 3.35±0.89 | 63.1% | 13.5% |
| 2-2-2 | 1 | 1 | 15 | -27.9±1.47 | 3.25±1.74 | 67.5% | 13.9% |
| 2-2-3 | 1 | 1.5 | 15 | -39.4±0.55 | 4.18±0.13 | 65.5% | 14.2% |
| 2-3-1 | 1 | 0.5 | 25 | -43.1±0.55 | 3.21±0.82 | 70.1% | 14.7% |
| 2-3-2 | 1 | 1 | 25 | -41.3±0.59 | 3.25±0.26 | 81.2% | 15.1% |
| 2-3-3 | 1 | 1.5 | 25 | -39.1±0.87 | 3.93±0.96 | 79.2% | 15.9% |
| 3-1-1 | 1.5 | 0.5 | 5 | -32.6±0.23 | 15.22±0.37 | 65.1% | 26.5% |
| 3-1-2 | 1.5 | 1 | 5 | -25.3±0.79 | 17.29±0.69 | 66.2% | 28.3% |
| 3-1-3 | 1.5 | 1.5 | 5 | -31.5±1.26 | 14.96±1.57 | 61.5% | 26.2% |
| 3-2-1 | 1.5 | 0.5 | 15 | -29.8±1.21 | 15.81±1.05 | 60.5% | 27.9% |
| 3-2-2 | 1.5 | 1 | 15 | -15.7±1.19 | 14.46±1.27 | 60.1% | 28.5% |
| 3-2-3 | 1.5 | 1.5 | 15 | -21.6±0.37 | 14.89±0.05 | 60.1% | 18.3% |
| 3-3-1 | 1.5 | 0.5 | 25 | -19.9±0.45 | 15.11±3.25 | 61.5% | 18.9% |
| 3-3-2 | 1.5 | 1 | 25 | -22.1±1.17 | 17.72±0.17 | 59.3% | 19.5% |
| 3-3-3 | 1.5 | 1.5 | 25 | -32.1±0.91 | 16.15±1.76 | 60.2% | 18.3% |

Sodium alginate-chitosan coated quercetin microspheres (SA-Q-CS MPs) were prepared by modified emulsifying crosslinking method with CaCl_2_ as the crosslinking agent. The effect of sodium alginate, chitosan and CaCl_2_ on zeta potential, particle size, entrapment efficiency and loading efficiency of microspheres were analyzed. The formulation variables and the high and low levels of each variable were defined based on preliminary experiments.
